# Radiological patterns and prognosis in elderly patients with acute *Klebsiella pneumoniae* pneumonia: A retrospective study

**DOI:** 10.1097/MD.0000000000029734

**Published:** 2022-08-12

**Authors:** Kosaku Komiya, Hiroki Yoshikawa, Akihiko Goto, Takashi Yamamoto, Mari Yamasue, Takeshi Johkoh, Kazufumi Hiramatsu, Jun-ichi Kadota

**Affiliations:** a Department of Respiratory Medicine and Infectious Diseases, Oita University Faculty of Medicine, Oita, Japan; b Department of Internal Medicine, Tenshindo Hetsugi Hospital, Oita, Japan; c Radiology, Kinki Central Hospital of Mutual Aid Association of Public School Teachers, Hyogo, Japan; d Department of Medical Safety Management, Oita University Faculty of Medicine, Oita, Japan; e Director, Nagasaki Harbor Medical Center, Nagasaki, Japan.

**Keywords:** elderly, pneumonia, radiology

## Abstract

Although *Klebsiella pneumoniae* pneumonia is an insidious threat among the elderly, the role of radiological features has not been elucidated. We aimed to evaluate thin-section chest computed tomography (CT) features and assess its associations with disease prognosis in elderly patients with acute *K. pneumoniae* pneumonia.

We retrospectively included elderly patients, admitted for acute *K. pneumoniae* pneumonia, and investigated thin-section CT findings to determine whether bronchopneumonia or lobar pneumonia was present. The association between the radiological pattern of pneumonia and in-hospital mortality was analyzed.

Eighty-six patients with acute *K. pneumoniae* pneumonia were included, and among them, the bronchopneumonia pattern was observed in 70 (81%) patients. Twenty-five (29%) patients died in hospital, and they had a greater incidence of lobar pneumonia pattern (40% in nonsurvivors vs 10% in survivors; *P* = .008), low albumin level (2.7 g/dL, range, 1.6–3.8 in nonsurvivors vs 3.0 g/dL, range, 1.7–4.2 in survivors; *P* = .026) and higher levels of aspartate aminotransferase (30 U/L, range, 11–186 in nonsurvivors vs 23 U/L, range, 11–102 in survivors, *P* = .017) and C-reactive protein (8.0 mg/dL, range, 0.9–26.5 in nonsurvivors vs 4.7 mg/dL, range, 0.0–24.0 in survivors; *P* = .047) on admission. Multivariate analysis showed that lobar pneumonia pattern was independently associated with increased in-hospital mortality (adjusted hazard ratio, 3.906; 95% CI, 1.513–10.079; *P* = .005).

In elderly patients with acute *K. pneumoniae* pneumonia, the lobar pneumonia pattern may be less commonly observed, and this pattern could relate to poor prognosis.

## 1. Introduction

*Klebsiella pneumoniae* is one of the most common Gram-negative bacteria that cause community-acquired pneumonia (CAP), accounting for 0.1% to 24.5% of all cases of pneumonia.^[1]^ With the aging of the population in many areas, the incidence of *K. pneumoniae* pneumonia among elderly people is gradually increasing and is a risk factor for severe CAP.^[2,3]^

*K. pneumoniae* pneumonia typically presents as lobar pneumonia characterized by inflammatory exudate within the intraalveolar space, resulting in lung lobe consolidation.^[4]^ However, pneumonia in elderly people is mainly acquired by aspiration of bacteria-containing oral secretions.^[5]^ We have reported that aspiration pneumonia, confirmed by the existence of swallowing dysfunction on videofluorography, is more prone to follow a bronchopneumonia pattern rather than lobar pneumonia pattern.^[6]^ In this regard, we hypothesized that *K. pneumoniae* pneumonia in elderly people would less commonly take a lobar pneumonia pattern, reflecting aspiration pneumonia. Furthermore, it is uncertain whether these radiological patterns affect disease prognosis in *K. pneumoniae* pneumonia. The objectives of this study were to analyze the frequency of the lobar pneumonia pattern among elderly patients with acute *K. pneumoniae* pneumonia and assess the correlation between radiological pattern and in-hospital mortality.

## 2. Methods

### 2.1. Patients and study design

This was a retrospective cohort study conducted at our hospital, a community hospital with 188 beds, in Oita Prefecture, Japan. In this study, CAP was defined according to the American Thoracic Society/Infectious Diseases Society of America guidelines,^[7]^ which comprised clinical signs and symptoms, including cough and fever, as well as infiltrates revealed by chest radiography or chest computed tomography (CT). Patients with hospital-acquired pneumonia and those treated with immunosuppressants were excluded from this study. We included consecutive patients (age ≥ 65 years) who had been admitted to the hospital between January 2015 and May 2021 for CAP caused by *K. pneumoniae* and who had undergone chest CT within 1 week before or after admission. Patients in whom bacteria other than *K. pneumoniae* were concurrently isolated from sputum were excluded, but those isolated as indigenous pathogens, not requiring specific antibiotics, were diagnosed as *K. pneumoniae* pneumonia. The study protocol was approved by the Institutional Ethics Committee of Tenshindo Hetsugi Hospital (Approval Number: 2040; Approval Date: April 12, 2021). Informed consent was waived by the committee because of the retrospective nature of the study. Some of the patients included in this study had already participated in previous studies.^[8,9]^

### 2.2. Data collection and evaluation of chest CT findings

Patient data on admission included sex, age, body mass index, and comorbidities. Laboratory data included white blood cell count, C-reactive protein (CRP) levels, albumin levels, and liver enzyme activity. These are usually routinely documented when a patient diagnosed with CAP is admitted. Sputum culture results were also collected from medical records. We defined respiratory failure as < 90% SpO_2_ without supplemental oxygen inhalation on admission. We evaluated daily physical activity, both before and on admission, using the Barthel Index. The Barthel Index was introduced in 1965 and originally used a 0 to 20 scale.^[10]^ It was modified by Granger et al in 1979 to include 0 to 10 points for each item (i.e., a total possible score of 0–100).^[11]^ Information relating to 10 basic activities of daily living is collected through the revised Barthel Index and includes bowel, bladder, grooming, toilet use, feeding, transfers, walking, dressing, climbing stairs, and bathing patterns.

A 320-detectors row CT scanner (AquilionONE; Toshiba Medical Systems) was used. Scans were obtained using 2.0-mm-thick sections of contiguous images from the apex to the base of the lung. Images were captured at a window setting of − 600 Hounsfield units (level) and 1500 Hounsfield units (width). If a patient had undergone CT before referral to our hospital, we evaluated the CT features from the images captured at the referring institute.

Two respiratory medicine specialists (with 16 and 17 years of experience, respectively, and who were blinded to the patients’ clinical information) retrospectively assessed the following chest CT findings: ground-glass attenuation (GGA), airspace consolidation, emphysema, reticular pattern, air bronchogram, bronchial wall thickening, centrilobular nodules, bronchiectasis, cavity, pleural effusion, and lymph node enlargement. They also determined the radiological pattern of pneumonia: bronchopneumonia or lobar pneumonia. Bronchopneumonia was defined as multiple opacities in a lobular pattern, along bronchus or bronchioles, whereas lobar pneumonia was defined as homogeneous opacification in a lobar pattern and sharply defined at the fissures.^[12,13]^ The distributions of GGA and/or airspace consolidation were also evaluated. The number of involved lobes was counted regarding lingular segments as one lobe.

### 2.3. Statistical analyses

Statistical analyses were performed using IBM SPSS version 24 software (IBM Japan). *P* < .05 was considered statistically significant. The kappa statistic was used to assess the concordance of imaging evaluations. Variables among patients’ backgrounds, laboratory data, presence of respiratory failure, and radiological patterns of pneumonia with a *P* value of < 0.05 in the univariate analysis were included in the multivariate analysis. To explain whether the radiological pattern was independent of other variables when used as a predictor of mortality, Cox proportional hazards regression was performed to evaluate the effect of radiological pattern on in-hospital mortality. Kaplan–Meier curves were constructed using the log-rank tests to compare the time to achieve the primary outcome between treatment groups.

## 3. Results

### 3.1. Baseline characteristics and thin-section CT features

Out of the 98 elderly patients who had been admitted to our institute for CAP and contained *K. pneumoniae* in their sputum cultures, 12 patients were excluded owing to concurrent isolation of bacteria other than *K. pneumoniae.* The remaining 86 patients had a chest CT within 1 week before or after admission and were therefore included in this study. Approximately 27% of patients were women, and the median age was 87 years, as shown in Table 1.

**Table 1:** Univariate analysis of the patients’ characteristics associated with bronchopneumonia/lobar pneumonia pattern in patients with acute *Klebsiella pneumoniae* pneumonia.

	All cases (n = 86)	Bronchopneumonia (n = 70)	Lobar pneumonia (n = 16)	Crude OR	*P*
Female	23 (27)	18 (26)	5 (31)	0.762 (0.233–2.491)	.652
Age (years)	87 (65–103)	87 (65–102)	87 (65–103)	0.995 (0.924–1.072)	.894
BMI (kg/m^2^)	17.2 (11.3–29.4)	16.7 (11.3–29.4)	18.4 (12.0–25.6)	0.934 (0.800–1.089)	.383
Impaired consciousness	23 (27)	19 (27)	4 (25)	1.118 (0.321–3.894)	.861
Barthal index before admission	5 (0–100)	5 (0–100)	5 (0–100)	1.004 (0.988–1.019)	.642
Barthal index on admission	0 (0–100)	0 (0–100)	0 (0–45)	1.023 (0.987–1.060)	.216
Systolic blood pressure (mm Hg)	122 (55–180)	123 (74–180)	114 (55–176)	1.015 (0.992–1.039)	.212
Respiratory failure	48 (56)	39 (56)	9 (56)	0.978 (0.327–2.924)	.969
Smoking history	45 (52)	38 (54)	7 (44)	1.527 (0.511–4.559)	.448
COPD	22 (26)	20 (29)	2 (13)	2.800 (0.583–13.455)	.199
Heart failure	15 (17)	12 (17)	3 (19)	0.897 (0.221–3.639)	.879
Cerebral vascular diseases	34 (40)	28 (40)	6 (38)	1.111 (0.363–3.403)	.854
Malignancy	7 (8)	6 (9)	1 (6)	1.406 (0.157–12.570)	.760
Diabetes mellitus	13 (15)	9 (13)	4 (25)	0.443 (0.117–1.674)	.230
Tube feeding	13 (15)	11 (16)	2 (13)	1.305 (0.259–6.564)	.747
WBC (/μL)	9405 (830–23,970)	9680 (2810–23,970)	7010 (830–1744)	1.000 (1.000–1.000)	.168
Hb (g/dL)	11.7 (6.4–16.1)	11.8 (6.4–16.1)	11.6 (7.1–15.5)	1.104 (0.827–1.475)	.501
Alb (g/dL)	2.9 (1.6–4.2)	2.9 (1.7–4.2)	2.8 (1.6–4.1)	1.093 (0.393–3.038)	.865
AST (U/L)	23 (11–186)	23 (11–102)	30 (11–186)	0.981 (0.960–1.003)	.087
eGFR (mL/min/1.73m2)	59.3 (8.4–200.8)	65.3 (9.1–200.8)	42.4 (8.4–193.9)	1.012 (0.996–1.027)	.149
CRP (mg/dL)	5.5 (0.0–26.5)	5.3 (0.0–26.5)	7.7 (0.7–24.3)	1.016 (0.936–1.104)	.699
ESBL positive	5 (6)	4 (6)	1 (6)	0.909 (0.095–8.728)	.934
Duration of hospitalization (days)	36 (0–177)	39 (0–177)	28 (0–129)	1.004 (0.986–1.023)	.628

The kappa values of the CT findings were as follows: 0.85 for GGA, 0.74 for airspace consolidation, 0.93 for emphysema, 0.55 for reticular pattern, 0.81 for air bronchogram, 0.68 for bronchial wall thickening, 0.88 for centrilobular nodules, 0.79 for bronchiectasis, 1.00 for cavity, 0.86 for pleural effusion, and 0.80 for lymph node enlargement. GGA, consolidation, centrilobular nodules, bronchial wall thickening, and pleural effusion were commonly observed, whereas reticular pattern, air bronchogram, and cavity were less common, as shown in Table 2. GGA and/or consolidation were mostly observed as bilateral.

**Table 2:** Thin-section chest computed tomography features.

	Frequency (%)
Radiological patterns	
Bronchopneumonia	70 (81)
Lobar pneumonia	16 (19)
Major features	
Ground-glass attenuation	83 (97)
Airspace consolidation	83 (97)
Distribution of GGA and/or consolidation	
Right upper	56 (65)
Right middle	48 (56)
Right lower	74 (86)
Left upper	43 (50)
Left middle	39 (45)
Left lower	74 (86)
Bilateral	
Number of lobe involvement	
Other features	
Emphysema	38 (44)
Reticular pattern	12 (14)
Air bronchogram	16 (19)
Bronchial wall thickening	76 (88)
Centrilobular nodules	39 (45)
Bronchiectasis	29 (34)
Cavity	4 (5)
Pleural effusion	45 (52)
Lymph node enlargement	24 (28)

On the basis of these CT features, radiological patterns of pneumonia were classified as bronchopneumonia in 70 cases (81%) and lobar pneumonia in 16 (19%). The kappa value for the radiological pattern of pneumonia was 0.86. Baseline clinical characteristics did not differ between bronchopneumonia pattern and lobar pneumonia pattern (Table 1).

### 3.2. Baseline characteristics and patterns of pneumonia in the nonsurvivor and survivor groups

**Figure 1.**
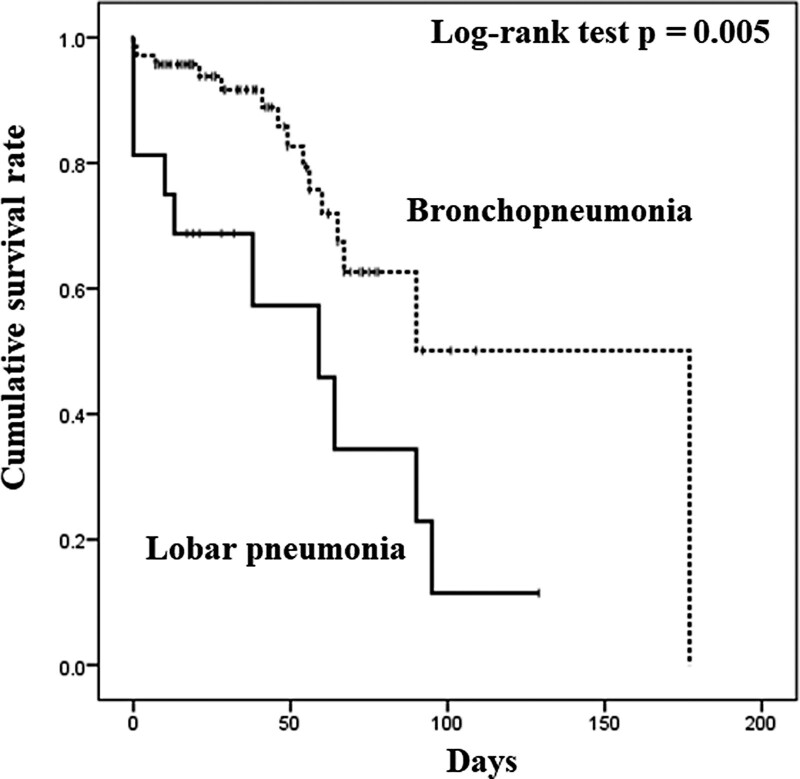
Kaplan–Meier survival probability based on the radiological pattern of pneumonia, bronchopneumonia (n = 70) or lobar pneumonia (n = 16), in elderly patients with *Klebsiella pneumoniae* pneumonia. Significant difference was observed in the overall survival between the groups.

In total, 25 (29%) patients died during hospitalization. Compared with the survivor group, the nonsurvivor group had a significantly higher incidence of lobar pneumonia as shown in Figure 1 (log-rank test, *P* = .005). Furthermore, the nonsurvivor group had lower albumin levels and higher levels of aspartate aminotransferase and CRP than the survivor group (Table 3).

**Table 3:** Univariate analysis of the patients’ characteristics associated with in-hospital mortality in patients with acute *Klebsiella pneumoniae* pneumonia.

	Nonsurvivors (n = 25)	Survivors (n = 61)	Crude HR	*P*
Female	7 (28)	16 (26)	0.972 (0.402–2.352)	.950
Age (years)	87 (78–102)	87 (65–103)	1.052 (0.988–1.121)	.112
BMI (kg/m^2^)	16.0 (11.3–25.8)	17.2 (12.5–29.4)	0.937 (0.802–1.094)	.410
Impaired consciousness	9 (36)	14 (23)	1.503 (0.654–3.458)	.337
Barthal index before admission	2.5 (0–100)	10 (0–100)	0.992 (0.981–1.004)	.209
Barthal index on admission	0 (0–25)	0 (0–100)	0.952 (0.902–1.005)	.073
Systolic blood pressure (mm Hg)	122 (55–166)	117 (74–180)	0.997 (0.979–1.015)	.742
Respiratory failure	14 (56)	34 (56)	0.860 (0.383–1.932)	.716
Smoking history	11 (44)	34 (56)	1.055 (0.468–2.378)	.897
COPD	3 (12)	19 (31)	0.413 (0.123–1.387)	.152
Heart failure	6 (24)	9 (15)	1.058 (0.392–2.856)	.911
Cerebral vascular diseases	8 (32)	26 (43)	0.859 (0.362–2.041)	.731
Malignancy	3 (12)	4 (7)	1.723 (0.506–5.868)	.384
Diabetes mellitus	3 (12)	10 (16)	0.277 (0.063–1.227)	.091
Tube feeding	4 (16)	9 (15)	0.951 (0.321–2.816)	.928
WBC (/μL)	8951 (830–15,090)	9410 (2810–23,970)	1.000 (1.000–1.000)	.243
Hb (g/dL)	11.5 (6.4–14.7)	12.0 (6.5–16.1)	0.895 (0.729–1.099)	.292
Alb (g/dL)	2.7 (1.6–3.8)	3.0 (1.7–4.2)	0.407 (0.184–0.899)	.026
AST (U/L)	30 (11–186)	23 (11–102)	1.016 (1.003–1.029)	.017
eGFR (mL/min/1.73m2)	45.2 (8.4–193.9)	67.3 (10.0–200.1)	0.993 (0.983–1.002)	.127
CRP (mg/dL)	8.0 (0.9–26.5)	4.7 (0.0–24.0)	1.058 (1.001–1.119)	.047
ESBL positive	1 (4)	4 (7)	0.628 (0.084–4.727)	.652
Lobar pneumonia/bronchopneumonia	10 (40)	6 (10)	3.068 (1.342–7.015)	.008
Bilateral	21 (84)	54 (89)	0.386 (0.127–1.176)	.094
Number of lobes	4 (1–6)	4 (1–6)	1.305 (0.985–1.729)	.064

We conducted a multivariate analysis using the variables that were significantly different between nonsurvivors and survivors in the univariate analysis. Results showed that only the lobar pneumonia pattern was significantly associated with in-hospital mortality, as shown in Table 4.

**Table 4:** Multivariate analysis of the patients’ characteristics associated with in-hospital mortality in patients with acute *Klebsiella pneumoniae* pneumonia.

	Adjusted HR	*P*
Alb (g/dL)	0.427 (0.182–1.001)	.050
AST (U/L)	1.003 (0.987–1.018)	.740
CRP (mg/dL)	1.045 (0.970–1.126)	.245
Lobar pneumonia/ Bronchopneumonia	3.906 (1.513–10.079)	.005

## 4. Discussion

This study showed that lobar pneumonia pattern was less commonly observed in elderly patients with acute *K. pneumoniae* pneumonia, and this radiological pattern was independently associated with in-hospital mortality.

Although *Klebsiella pneumoniae* pneumonia generally shows a lobar pneumonia pattern, this study showed inconsistent results. One possible reason for this discrepancy is that the current study focused on elderly patients, and most cases were characterized by aspiration pneumonia. Aspiration pneumonia develops through the accumulation of microaspirations,^[14]^ and ongoing microaspirations fall into a bronchopneumonia pattern. In fact, we previously studied the chest CT features in patients with aspiration pneumonia confirmed by videofluorography, with the results showing that most cases exhibited the bronchopneumonia pattern.^[6]^ Furthermore, bronchopneumonia could progress to lobar pneumonia over time. In this regard, most patients had easy access to medical care and were characterized with bronchopneumonia as an early phase of pneumonia.

In the current analyses, the lobar pneumonia pattern was significantly associated with increased in-hospital mortality. In general, the proposed risk factors for mortality in CAP include advanced age, physical activity level, albumin level, inflammatory markers, and respiratory status.^[15–18]^ Interestingly, even after adjusting for these variables, the lobar pneumonia pattern was an independent predictor for poor prognosis. Considering that the number of involved lobes was not associated with in-hospital mortality, it appears that the extent of pneumonia does not simply affect disease prognosis. Lobar pneumonia is characterized by inflammatory exudate within the intraalveolar space, which might reflect overreactive immune responses in the host.^[19]^ These host reactions could explain the poor prognosis among patients with lobar pneumonia patterns. In addition, as previously stated, bronchopneumonia may progress to lobar pneumonia. Although we were unable to estimate the time interval from disease onset to chest CT imaging because the onset in elderly people is unclear, cases of lobar pneumonia might have been diagnosed at a late stage of pneumonia development.

Meanwhile, apart from host reactions, there is a need to discuss *K. pneumoniae* virulence. In the 1980s, extended spectrum beta-lactamase (ESBL)–producing *K. pneumoniae* was discovered, which has gradually spread worldwide.^[20]^ ESBL-producing *K. pneumoniae* has reached a prevalence rate of 50% in several areas globally, as reported by the World Health Organization.^[21]^ Carbapenem represents the first-line therapy for severe infections caused by ESBL-producing *K. pneumoniae*. However, carbapenemase-producing *K. pneumoniae* was isolated in 1996,^[22]^ and the strain is becoming a highly threatening pathogen, especially in a nosocomial setting.^[23]^ Treatment options are extremely limited for these resistant strains, and infected patients have a higher mortality than those with a nonresistant strain.^[24]^ At our institute, screening for such resistance was not routinely performed, so it is unknown whether these potentially resistant strains affect disease progression. If they do, resistant strains might influence or confound the pattern of pneumonia.

To the best of our knowledge, this study is the first to demonstrate the association between the radiological pattern but not a simple extension of pneumonia, and disease mortality. However, this study has some limitations. First, *K. pneumoniae* may colonize the oral cavity or laryngopharynx in some cases^[20]^; therefore, it is technically impossible to exactly discriminate infection from colonization. Quantitative sputum culture or evaluation of Gram staining of sputum might provide useful information. Second, although we suggest that aspiration pneumonia can explain the bronchopneumonia pattern in elderly people, the swallowing function of patients included in this study was not routinely evaluated. We previously demonstrated that chest CT in patients with swallowing dysfunction showed a bronchopneumonia pattern, but it remains unclear whether this pattern is highly specific for diagnosing aspiration pneumonia because no comparison study with patients with normal swallowing function has been performed. Finally, investigator agreement on radiological features was not perfect, as represented by the kappa values.

In conclusion, the lobar pneumonia pattern in elderly patients with acute *K. pneumoniae* pneumonia appears to be an independent risk factor of in-hospital mortality. A larger study is required to validate this finding, evaluate the correlation between swallowing function and patterns of pneumonia and screen for drug-resistant strains, and provide an effective treatment strategy depending on the pneumonia pattern.

## Acknowledgments

The authors thank Dr Kenji Umeki and Dr Eiji Okabe for clinical advice.

## Author contributions

K. K., H. Y., K. H., and J. K. designed this study and drafted the article. K. K., H. Y., A. G., T. Y., M. Y., T. J., K. H., and J. K. contributed to the data collection, data analysis, and helped draft the article.
